# Tumors of the Mouth

**Published:** 1895-08

**Authors:** C. G. Darling

**Affiliations:** The Dental Department of the University of Michigan


### THE DENTAL REGISTER.

VOL. XLIX.] AUGUST, 1895. [No. 8.

### COMMUNICATIONS.

### Tumors of the Mouth.

BY DR. C. G. DARLING, M.D. OF THE DENTAL DEPARTMENT OF THE UNIVERSITY OF MICHIGAN.

Read before the Mississippi Valley Dental Society, April 18th, 1895.

Tumors small and benign give neither the patient nor the dentist much concern. They are early submitted to him for treatment and he usually brings them to a successful termination, but the malignant varieties coming insiduously, never giving a hint of their true character until they are decidedly present, already appropriating space and tissue to their own use and like the squatter, determined to hold the ground at any cost, they soon become a source of danger and discomfort to the patient, exciting the anxiety of the dentist, and wise is he who at an early date seeks refuge in consultation in all cases where the malignancy of the growth may be questioned.

It is not the object of this paper to treat of the origin and pathology of those tumors which may appear in the mouth but rather to examine them from a surgical standpoint and discuss methods of surgical treatment.

Fortunately the risk to life in operations about the face and mouth is slight when compared with operations of the same magnitude in other parts of the body. No life-sustaining organs are involved. Shock is not great and sepsis is so slight in wounds of this locality that it seldom proves fatal. Therefore nearly all operations about the face are a success so far as recovering from the effects of the operation and the repair in tissue may be concerned. Even those failures made so by a return of the disease are not without good results, and I will attempt to show by the records of a few cases, that even the unpromising and rapidly developing tumors may be removed, prolonging the life of the patient and giving comfort in his last days.

The first case was treated by Prof. Nancrede in the university clinic and has been reported by him in the "*Annals of Surgery.*"

E. L., a man thirty-three years of age, entered the University Hospital in March, 1891, and gave the following history: In July of 1890 he had noticed a small growth on the inferior border of the lower jaw which corresponded to the position of the first molar tooth. Early in October two teeth were extracted (whether by physician or dentist I do not know), under the impression that at the roots of these would be found the cause of the trouble and that it could be easily removed, but, instead of improvement, there sprung up from the alveolar process a mass which rapidly increased in size until nearly all of the right half of the lower jaw was involved. The growth soon began to break down, while the discharges and sloughs from this suppurating mass were not entirely expelled but portions were swallowed and taken into the lungs causing a rapid decline in the patient's health. The diagnosis of rapidly growing sarcoma was made and on March 30, 1892, the first operation was made; removing a little more than half of the lower jaw. The parts healed promptly and in three weeks the patient left the hospital apparently cured.

Three weeks later, however, he returned with the disease well advanced in the remaining portion of the jaw. This was promptly removed together with the floor of the mouth well down to the base of the tongue. June 10, 1892, he again returned to his home and no change for the worse was noticed for two months, when nodules began to develop on the right side near the old scar. These continued to increase in size and join together until quite a bulky tumor was formed. For the third time he came to the hospital; again it was decided that operation might give relief, although the tumor now extended well up toward the temporal region involving nearly all branches of the facial nerve and reaching downward along the carotid vessels. After a tedious and dangerous dissection which lasted more than two hours the growth was successfully removed.

Again the patient made a good recovery and died about two years later from the effects of sarcoma in the lungs and kidneys. During this time he was remarkably free from pain and seemed to enjoy life. No great deformity was caused by this extensive operation and he could speak nearly as distinctly as before, though the entire lower jaw and the floor of the mouth were removed.

The next case (also in Prof. Nancrede's clinic) while less formidable than the preceding one, was made so by the character and extent of the growth, the patient having submitted to the operation while the disease was probably confined to a portion of the lower maxillary bone.

Mr. C. E., thirty-seven years old, had always enjoyed excellent health. Twelve years ago the appearance of the right lower wisdom tooth gave him much trouble and it became necessary to extract it.

During the operation a portion of the root was broken off and allowed to remain. From this time the parts were sensitive and easily irritated and a few weeks later a small tumor developed which was probably cystic or soon became so, for, he says, it was opened many times by physicians and dentists always discharging a thin water-like fluid but never discharging any pus.

Six years ago it began to increase in size but made slow progress until about two months ago, when a change came on rapidly and at the time he entered the University Hospital, March 14, 1895, the growth had extended forward on the lower jaw nearly to the median line, backward to the angle and well up the ramus, pushing the tissues of the cheek prominently outward. There was an opening in the central portion of the tumor from which flowed a thin offensive fluid; the patient stated that some small pieces of bone had been discharged through the opening, but no trace had been discovered of the offending root. All the molar teeth on that side have recently been extracted and their place is now occupied by the growth.

A probe inserted into the opening revealed the fact that some fragments of bone still remained, which would undoubtedly come away soon if the tumor is allowed to pursue its course unmolested.

Excepting these, a large portion of the bone seems to be destroyed or displaced by the growth.

Excision of the right half of the lower jaw together with the outlying parts of the growth offered the best chance for a cure, and the patient promptly selected this course.

The operation did not present any unusual points of difficulty except that the bone was easily broken at the point of disease making it much more difficult to remove the ramus.

Recovery has promptly taken place and at this time he is well. In this case is found, what was at first a very simple tumor, a dentigerous cyst or epulic growth, after many years of irritation changing to a sarcoma. The dread which the physician, dentist or patient may have of operation, or their faith in medical measures probably were responsible for the delay which had allow this change to take place. This case is an earnest appeal to early and complete removal of all tumors of the mouth except in those cases where the growth is not irritated, remains stationary, and can be frequently seen by the surgeon or dentist in charge, and operation advised, when the slightest change for the worse takes place.

Sarcomatous tumors of the jaw may be removed with a reasonable hope of cure when the growth is central (myeloid sarcoma), if the entire bone to which the disease is confined be removed. Periosteal sarcoma does not promise such good results, because the surrounding tissue may be involved, but these tumors come early in life when the patient is vigorous and will rapidly recover from operations. In all tumors of this class, not only the jaw should be removed to the median line, but the entire periosteum and attachments of muscles must also be taken away, if we operate with any expectation of a cure. Another tumor of the mouth remarkable because of its slow growth and change, occurred in a patient who entered the University Hospital in June, 1894, during my term of service in the clinic.

Mrs. D., sixty-six years old, was for many years troubled with catarrh. Twelve years ago she first noticed a growth in the hard palate to the left of the median line. She was wearing a plate at the time and believed that it may have irritated the mouth and thus caused a tumor to develop, at least the irritation was soon so great that she could not wear it.

The tumor probably began in the hard palate or the floor of the left antrum, was a very slow growth until a few months since when it began to rapidly increase in size. It involved all the border of the left maxilla, nearly all of the hard palate and much of the soft palate. The left antrum and the nasal passages were filled by the extension of the growth upward. It projected so far downward that the mouth could scarcely be closed. An exploring needle passed into the tumor did not come in contact with bone where the border of the maxilla and the hard palate should be found. A portion of the tumor was excised for microscopical examination and the pathologist reported carcinoma.

The malignant nature of the tumor and its certain fatal result if allowed to remain, together with the danger of a return of the disease if the tumor was removed, were all explained to the patient. She expressed a strong desire to have the operation made and it was done, Prof. Nancrede kindly assisting me. Tracheotomy was first performed and the larynx packed with a sponge, that we might continue the anæsthetic and the operation at the same time without having blood enter the trachea, as it certainly would with the severe hemorrhage we must encounter unless such means were taken to prevent it. An incision was carried from the middle of the lip upward to the left of the nose and under the left eye, the flap was then reflected to give sufficient room for cutting the bone. The alveolar portion was first divided in the median line, then the molar and nasal portions, after which the diseased part was torn from its resting place by the lion forceps. The alveolar border was cut away at least one inch to the right of the median line and all the surface from which the growth was removed was scraped with a curette to remove any small particles of diseased tissue that might be remaining. Hemorrhage was stopped by means of the Paquelin cautery, and the cavity was packed for forty-eight hours with iodoform gauze.

The patient made a good recovery and returned to her home in a few weeks, free from pain and relieved of the presence of such a tumor. When last heard from she was entirely well. The disease in this case may return but not for some months. During this time she will be free from pain and death may result from some other disease.

Here is another case illustrating the proper method of operating for carcinoma when the surgeon sees his case early.

Mr. W. H., farmer, has been a moderate drinker, enjoying good health. Three weeks ago he first noticed a small growth on the under surface of the right side of his tongue, it was closely connected with the surrounding tissue, growing rapidly and painlessly. A small portion was excised under cocaine anæsthesia and submitted to microscopical examination. October 17, 1894, he was operated upon in Prof. Nancrede's clinic, one-half of the tongue being removed well back to the base. Rather a heroic operation for a small growth of three weeks duration you may say, but here is the pathologist's report: "I have made sections of the growth from the tongue already reported as epithelioma and as far as I can see you have gone well outside of the disease." This extensive operation was the only hope of cure for this man, even then the lymphatic glands may have been involved, and the return of the disease may be noted at that point while the scar surface remains healthy. Cancer appearing in the mouth can scarcely be mistaken for any other growth, and there is never any difficulty in removing a portion to confirm the diagnosis, then prompt and thorough removal is the only chance for cure. When any lymphatics are found to be suspiciously enlarged, they must also be removed at the same time.

Mechanical devices to take the place of parts removed or to correct deformities following operations for malignant disease should not be applied for some time after complete recovery because of the danger that such irritation might renew the disease.

When the cicatrices are well formed or the diseased condition is not malignant nor beyond the bone, this point may be disregarded and the deformity corrected. The use of medicine in the treatment of malignant tumors is not to be considered where operation is possible but may be tried as a last resort. Coley, of New York, has recently reported thirty-five cases treated by injecting the combined toxines of erysipelas and the bacillus prodigiosus. In five of these cases he has reasonable hope of a permanent cure. All of his cases were inoperable and the diagnosis was verified microscopically. The investigations being carried on at the present time concerning the causes and treatment of these tumors certainly promise great results.

While we wait for these promises to become truths, let early and complete operation be the leading treatment of all tumors of the mouth, then few of our patients can say—

"Oh that comfort comes too late,
Tis like a pardon after execution;
That gentle physic given in time had cured me;
But now I am past all comfort here, but prayers."

DISCUSSION—AFTERNOON SESSION.

Convention met, pursuant to adjournment, President Taft in the chair.

The Chair announced that the discussion on Dr. Darling's paper, read at the close of the morning session, would be next in order.

Dr. Fletcher: I was one of those appointed to discuss the paper, and I would say that there is a great deal that might be presented on the subject, provided I knew enough about it. Personally I do not know much about tumors, and their treatment. Nevertheless, I have had sufficient experience in removing a few of them, and their after treatment to be aware that it is an excedingly difficult matter to entirely eradicate even a benign tumor at times. I have this morning seen a patient, who, within the past thirty days, has had a part of the superior maxillary bone, on the right side, removed, for a malignant growth of some character. The patient came from the northern part of the State, for the purpose of consultation, and amongst others he called upon me. He was told by four or five of our best surgeons that the probabilities were that the tumor would return. On examining the cicatrix, as far as it had formed, there was an appearance about the edge, of an angry, red, congested condition which indicated, even to one of limited experience, that there was still trouble brewing. The probabilities are, that before many months, or a year or two at the farthest, that the tumor will return. This coincides to some degree with the statements of the paper, as they were most of them what are regarded as complete cures for the time being. However, I believe he states finally that a true carcinoma is not likely to be cured permanently by any treatment you can give it. As far as my knowledge of those things goes, the benign tumors or bony tumors, are of a different character, may be and often are permanently removed. A point that we should probably be more interested in as specialists is that of the etiology of this character of tumors. I hold in my hand a specimen of which I will read you the clinical history. This case was operated on by Dr. P. S. Connor, of our city. He reported the case to me as follows: dated November 3d, 1894. Miss M. (colored,) age 26 years. Since the age of 5 years has had a small lump in the jaw, which grew very slowly until about six months ago. Jaw removed November 6th, 1894. Extended wound; healed by first intention; discharged November 20th, 1894; well. That, of course, as you may see by the extent of the tumor, and the amount of tissue taken away, would leave the face in a very bad shape. No doubt we would all like to consider ourselves as dental surgeons; but it is beyond the limits of legitimate dentistry and stomatology to go into this; such cases more properly belong to a surgeon, who is thoroughly fitted in every way, experience, knowledge and instruments, for this character of work. However, I consider it our duty to know enough to see upon examination of the mouth when it needs such attention. To be sure, a tumor of this character and size is plain enough for any body to see.

Ten days ago I took from the left side of the lower jaw, a small tumor, which has the characteristics, as far as I am able to tell, of a malignant growth; but I have not yet made an examination of it. It seems to me these tumors must have an exciting cause, and these causes may in there origin be connected with the pathology of the teeth. They may arise from the extraction of teeth. This case was sent to me after a week's suffering from an extracted lower molar. There was intense inflammation of the jaw about the seat of extraction. The bone about the sockets of the teeth was highly inflamed, and the soft tissues were exceedingly tender. I took this, at first, to be a matter of poisoning from unclean forceps. Whether such was the fact or not I do not know. In the treatment of the case, before it healed, a tumor showed itself just posterior to the sockets; and ten days after taking this tumor off I found it one-half the size it was before. That is about ten days ago. It is now nearly time for it to return again. If I still find that growing, that is, if it has started again, I shall feel considerably exercised over the man's future. He is a strong healthy man, thirty-five years of age, and has every condition about him to fasten a malignant tumor, as far as I could see. This bony tumor removed by Dr. Connor is of special interest, from the fact that a molar tooth, superior wisdom, has been carried clear out of its place, the crown of it is directed towards the nose, about the junction of the vertical plate of the palate bone where it joins on to the superior maxillary. You can see, Mr. President, that it is entirely out of place. This condition, here, I consider the exciting cause, (showing the place) as I explained before another Society where this was discussed. I found between the teeth, where the tumor had been sawed in two, a membrane going in from the mouth, showing that irritation of some character probably started at that place. My theory is that, from some dental lesion there was sufficient irritation of the periosteum on the floor of the antrum to start a growth in that particular position. As the history, which I read, shows, this tumor started at about five years of age. At that time the dental follicle holding the wisdom tooth was far enough back, and far enough up in the antrum, (a point which I expect to explain in a paper which is to follow) to undermine that follicle, taking with it the bone that surrounded it, and making a complete circuit, almost; coming around until the crown of the tooth points towards the nose; and it can be accounted for in my mind only in that way; this tumor, starting at that point, simply carried with it the dental follicle and its surrounding tissues, and pushed it about the antrum until it has finally landed where you will see it on examination. This case is very interesting to us, because of this particular feature of exciting causes which may arise from teeth. These exciting causes may be old roots of teeth, salivary calculus, alveolar abscess, in fact anything that continually irritates the tissues in those localities; so that it behooves us as a profession to look after these things, and be ready when there is a semblance of cause of that kind of trouble, to remove it. The rough edges of worn teeth, broken teeth, everything of that character that can irritate the mucous membrane, may cause a malignant growth.

Further than this, Mr. President, I believe I have nothing to say on the subject of tumors.

PRESIDENT TAFT: Are there any further remarks on this topic? Has any one any questions, or suggestions, to make? Can we hear from you, Dr. Respinger?

DR. RESPINGER, of Geneve, Switzerland: There are more competent men to speak on that subject than myself. I have not any experience in that direction.

PRESIDENT TAFT: Then we will proceed to the next order of business unless there is some one else who wishes to be heard.

DR. CALLAHAN: I move we have Mr. Fletcher's paper, and let anything further in the line of tumors be discussed at the close of that paper.

[*To be continued.*]

---

SO STRANGE.—"You say that I was born in Berlin, papa? Well, then where was mamma born?

"In Magedeburg."

"And where were you born, papa?"

"In Hanover, my child."

"Isn't it strange that we three got to know each other?"

